# Pre-malignant conditions diagnosed following a positive cancer signal from a multi-cancer early detection test

**DOI:** 10.3389/fonc.2024.1461693

**Published:** 2024-10-25

**Authors:** Omair A. Choudhry, Angana B. Kharge, Seema P. Rego, Paul Z. Elias, Adam H. Buchanan, Anne Marie Lennon, Nickolas Papadopoulos, Frank Diehl, Tomasz M. Beer

**Affiliations:** ^1^ Exact Sciences Corporation, Madison, WI, United States; ^2^ Geisinger, Danville, PA, United States; ^3^ Department of Medicine, University of Pittsburgh, Pittsburgh, PA, United States; ^4^ School of Medicine, Johns Hopkins University, Baltimore, MD, United States

**Keywords:** multi-cancer early detection, screening, early detection, CancerSEEK, DETECT-A, outcome

## Abstract

Blood-based tests for multi-cancer early detection (MCED) are being developed to facilitate the detection of various cancer types. The Detecting cancers Earlier Through Elective mutation-based blood Collection and Testing study (DETECT-A) study evaluated an MCED test in 9,911 women, age 65-75, without personal history of cancer. In a *post-hoc* analysis, we report on the detection of precancerous neoplasms consequent to MCED testing and follow-up. Participants with positive baseline and confirmatory MCED testing underwent 2-deoxy-2[fluorine-18] fluoro-D-glucose positron emission tomography-computed tomography (PET-CT) and diagnostic evaluation as indicated by PET-CT results. We reviewed the electronic health records of participants with a precancerous neoplasm and summarized their clinical course. MCED results were positive in 134 participants. Clinically significant pre-malignant conditions were identified in three of these participants: A 71-year-old with an ovarian mucinous cystadenoma, a 67-year-old with an appendiceal mucinous neoplasm, and a 70-year-old with colon adenomas displaying high-grade dysplasia. All three participants underwent surgical treatment and remain alive and cancer-free as of last follow up. The diagnostic evaluation of a positive MCED test may occasionally reveal clinically significant pre-cancerous conditions amenable to interventions. The frequency of such findings and their clinical impact warrants further study.

## 1 Introduction

Blood-based MCED tests have the potential to detect multiple types of cancer, including uncommon cancers and those that lack standard-of-care screening options. Similar to standard-of-care cancer screening tests, a positive MCED test result provides a suspicion of cancer that requires a diagnostic evaluation to establish a definitive diagnosis. Several prospective studies investigating the clinical performance of MCED testing are underway. However, there are limited published data describing the diagnostic journey and outcomes of patients with positive MCED tests and pre-malignant findings following a clinical workup.

The DETECT-A study was the first prospective interventional trial to assess an MCED test, CancerSEEK, a multi-analyte assay that evaluated circulating proteins and cell-free DNA (cfDNA) mutations in the blood (1).

DETECT-A participants with positive baseline and confirmatory CancerSEEK tests underwent subsequent PET-CT with follow-up procedures, when clinically indicated, to confirm the presence and location of cancer.

Out of 134 participants with a positive CancerSEEK test, 26 were diagnosed with cancer. Of the 108 false-positive cases, 7 were adjudicated as false positive without PET-CT imaging, 98 were classified as false positive following PET-CT imaging, and three were diagnosed with premalignant conditions and are the focus of this report.

## 2 Methods

DETECT-A was an ongoing prospective, interventional study to evaluate the performance of CancerSEEK, an early version of the Exact Sciences MCED test currently in development. The study included 10,006 women 65 to 75 years of age with no personal history of cancer (1). The study was approved by the Institutional Review Boards (IRBs) for Human Research at the Geisinger Health System (Geisinger; #2017-0268) and the Johns Hopkins Medical Institutions (#00119844) and was compliant with U.S. Common Rule and The Health Insurance Portability and Accountability Act. Written, informed consent was obtained from all participants. Women with a current or previously known cancer were excluded from the study.

The DETECT-A study utilized a two-step MCED testing process for determining CancerSEEK positivity prior to a subsequent diagnostic evaluation. The multi-analyte CancerSEEK test incorporated the analysis of specific somatic mutations in cell free DNA (cfDNA) and the detection of nine cancer-associated protein biomarkers.

The cfDNA mutations were assessed using a sequencing error reduction technology (2) that was comprised of a panel designed to cover common oncogenic hot spot regions of 16 genes (represented by 61 amplicons). While specific mutations were considered when determining positive test results, there were no strict criteria established to differentiate between pathogenic variants and variants of uncertain significance (VUS). Therefore, the panel had the capacity to detect both known pathogenic variants and those of uncertain significance. The nine protein biomarkers included CA15-3, CEA, CA125, CA19-9, HGF, AFP, OPN, TIMP-1, and Prolactin.

A CancerSEEK confirmation test was performed only on participants with a positive baseline test. It employed the same sequencing error reduction technology and the high cfDNA mutation and protein biomarker thresholds used in the baseline test, but assessed only the specific cfDNA mutations or proteins that were abnormal in the baseline test. It also rigorously excluded cfDNA mutations attributed to clonal hematopoiesis of indeterminate potential (CHIP) through more thorough examination of a larger amount of white blood cell (WBC) cfDNA than what was used in the baseline CancerSEEK test. Technical details of the baseline and confirmation test components are provided in the Lennon, et al., study (1). A multidisciplinary review committee then reviewed the medical histories of cases with positive baseline and confirmatory CancerSEEK tests to rule out potential non-cancer-related conditions that could potentially result in a positive Cancer SEEK result.

## 3 Results

### 3.1 Participant 1

**Figure 1:**
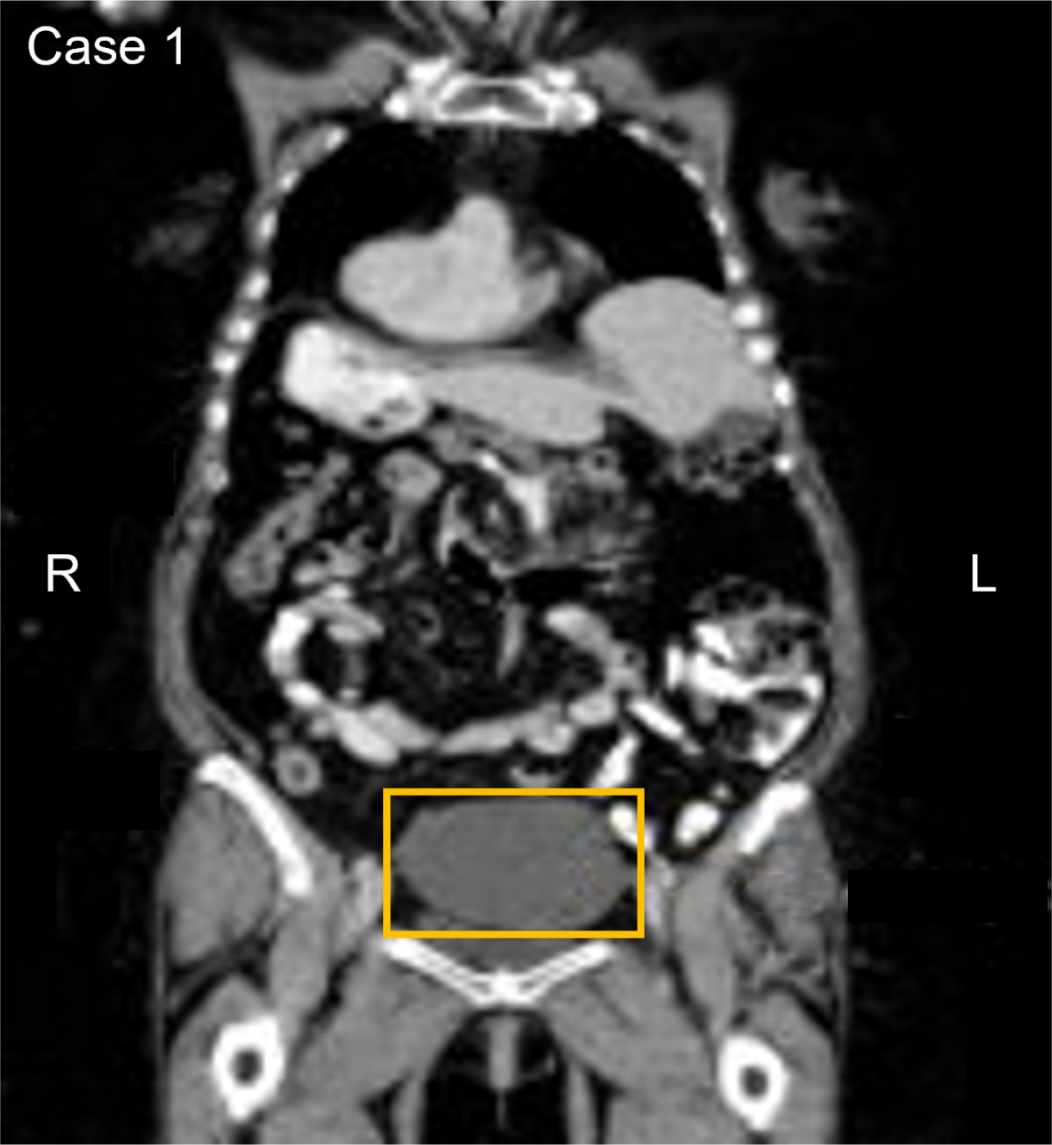
Participant one CT image. The area in the yellow box shows a large cystic mass in the pelvis measuring 10.3 x 9.8 x 7.8 cm.

A 71-year-old asymptomatic participant with no identified comorbidities tested positive on the baseline and confirmatory CancerSEEK tests based on a substitution in the *PIK3CA* gene, NM_006218.4(PIK3CA):c.1030G>A (p.Val344Met) (**Table 1**). The Variant Allele Frequency (VAF) for the *PIK3CA* substitution was 1.146% in the baseline test and 1.36% in the confirmatory test. PET-CT imaging identified multiple pulmonary nodules accompanied by calcified granulomas and mild bronchiectasis, and a pelvic cystic mass. Bilateral lung nodules measuring 16, 11, 8, and 5 mm in greatest diameter were noted. The lung nodules remained stable on CT monitoring and were assessed to be inflammatory, rather than malignant. The pelvic mass, measuring 10.3 x 9.8 x 7.8 cm (**Figure 1**), was similarly monitored, with the patient eventually undergoing a laparoscopic left oophorectomy and salpingectomy 15.7 months after initial CancerSEEK testing, revealing a mucinous cystadenoma. The tissue was not available for mutational analysis. The participant remained alive and cancer-free 3 years and 5 months after enrollment, which marks the conclusion of participation due to voluntary withdrawal from study.

**Table 1:** Details of pre-cancerous findings detected by the CancerSEEK MCED blood test.

	Baseline and Confirmation Test Result/ClinVar variant ID	Imaging Performed	Additional Procedures	Pre-malignant Condition	Treatment	Time from test to surgical intervention	Outcome as of last follow up
**Participant 1**	DNA (*PIK3CA*) chr3 178921548 G>A (NM_006218.4):c.1030G>A (p.Val344Met)	PET-CT	CT Scan Lung (Pulmonary Nodules)	Benign ovarian mucinous cystadenoma	Surgery- Laparoscopic Left Oophorectomy and Salpingectomy	15.7 months	Alive and Cancer-free (08/2021)
**Participant 2**	DNA (*TP53*) chr17 7578265 A>G (NM_000546.6):c.584T>C (p.Ile195Thr)	PET-CT	Colonoscopy	Carcinoma *in situ* of the appendix	Surgery- Laparoscopic appendectomy and partial cecectomy	16.6 months	Alive and Cancer-free (02/2023)
**Participant 3**	DNA (*KRAS*) chr12 25398284 C>T NM_004985.5(KRAS):c.35G>A (p.Gly12Asp)	PET-CT	Colonoscopy	Colonic adenomas with high grade dysplasia	Surgery-Right hemicolectomy	12.5 months	Alive and Cancer-free (02/2023)

PET-CT, 2-deoxy-2[fluorine-18] fluoro-D-glucose positron emission tomography-computed tomography; CT, computed tomography.

### 3.2 Participant 2

**Figure 2:**
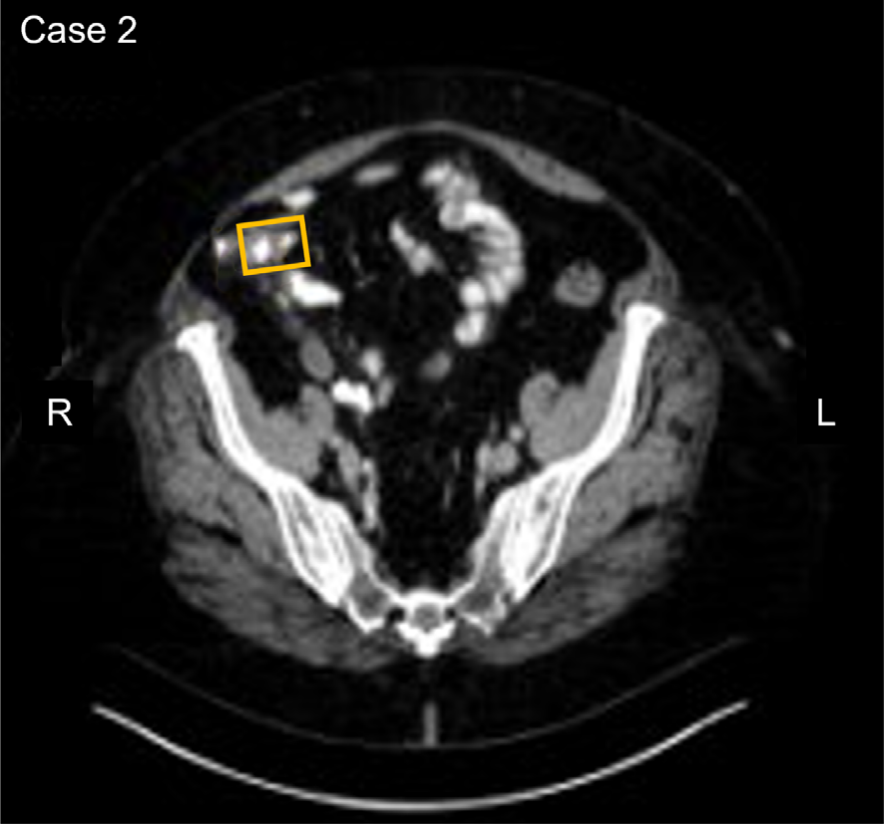
Participant two CT image. The area in the yellow box shows an appendix measuring 1.3 cm and displaying internal fluid density without surrounding inflammatory changes.

An asymptomatic 67-year-old participant with a history of connective tissue disease, tested positive on the baseline and confirmatory CancerSEEK tests based on a substitution in the *TP53* gene, NM_000546.6(TP53):c.584T>C (p.Ile195Thr) (**Table 1**). The variant allele frequency for the *TP53* substitution was 0.06% in the baseline test and 0.018% in the confirmatory test. PET-CT imaging revealed an appendix measuring 1.3 cm in diameter and displaying internal fluid density without surrounding inflammatory changes, peripheral calcification, associated 18F-FDG uptake, or lymphadenopathy (**Figure 2**). Based on the PET-CT findings, a colonoscopy was performed, revealing a 3 mm benign sessile polyp, which was excised. 16.6 months after enrollment, the participant underwent laparoscopic appendectomy and partial cecectomy, revealing a 7.9 x 1.8 cm appendix. Histopathological assessment revealed the presence of a non-invasive low-grade appendiceal mucinous neoplasm (LAMN). This participant was adherent to standard of care (SOC) colorectal cancer screening (FOBT test) within 12 months after their first blood draw, and the FOBT test was negative. The tissue sample was negative for the *TP53* variant. The participant remains alive and cancer-free at last follow-up, 5 years and 6 months after enrollment.

### 3.3 Participant 3

**Figure 3:**
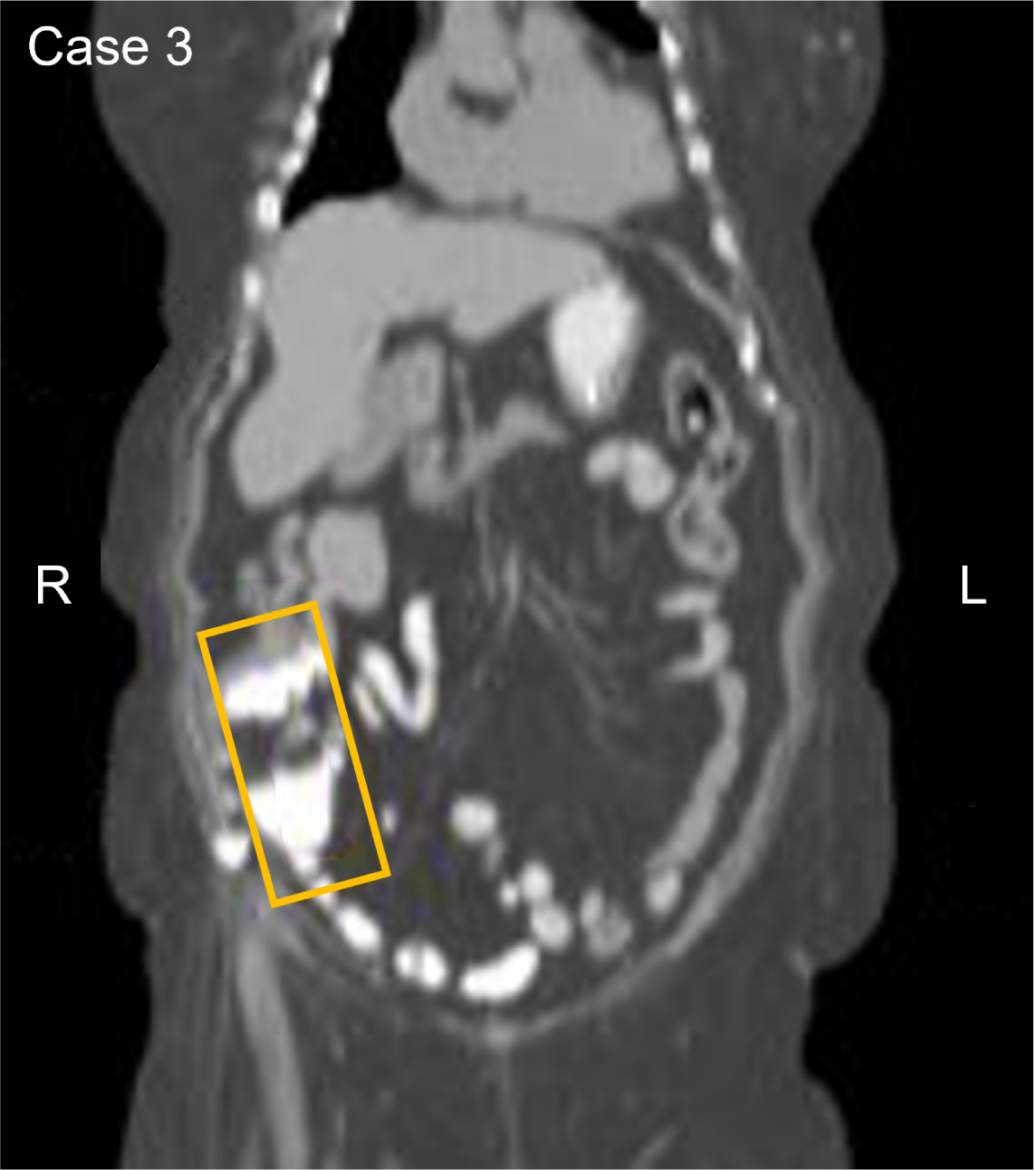
Participant three CT image. The area in the yellow box shows increased metabolic activity in the cecum and ascending colon with a filing defect suggesting a possible anomaly or irregularity in this region.

An asymptomatic 70-year-old participant with a history of moderate to severe renal disease, tested positive on the baseline and confirmatory CancerSEEK tests based on a substitution in the *KRAS* gene, NM_004985.5(KRAS):c.35G>A (p.Gly12Asp) (**Table 1**). The baseline test detected a *KRAS* substitution at a VAF of 0.078% and 0.047% in the confirmatory test. PET-CT imaging identified a filling defect and increased metabolic activity in the cecum and ascending colon (**Figure 3**). Colonoscopy identified 2 masses, one at the ileocecal valve and the other in the ascending colon. Additionally, 5 small polyps (tubulovillous adenomas and hyperplastic polyps, including one with mucosal Schwann cell hamartoma) (3) were removed at colonoscopy from the rectum and sigmoid colon. The hamartoma represents an exceedingly rare neurogenic tumor (4), with only 35 cases reported to date, all of which were incidentally found during colonoscopy (5). The patient was evaluated for endoscopic resection, which was not deemed feasible. A right hemicolectomy was therefore performed 12.5 months following enrollment, revealing a 4.5 cm tubulovillous adenoma with focal high-grade dysplasia at the ileocecal valve, a 5 cm tubulovillous adenoma with similar dysplasia in the ascending colon, and a 0.7 cm tubular adenoma in the ascending colon. This participant was adherent to SOC colorectal cancer screening; however, the details of the SOC testing were not available. The adenoma tissue was not available for mutational analysis. The participant remains alive and cancer-free at last follow-up, 5 years and 1 month after enrollment.

## 4 Discussion

Understanding non-cancer findings that may become evident in the course of a diagnostic evaluation of a positive MCED result will aid clinicians evaluating such patients. Fewer than 3% of participants with positive CancerSEEK results in DETECT-A had pre-cancerous lesions identified. In all three cases, the triggering biomarker was a DNA variant, the diagnoses represented clinically significant conditions, and were judged by clinicians to warrant therapeutic intervention to remove the lesions.

The diagnosis of the ovarian mucinous cystadenoma was prompted by the detection of a *PIK3CA* variant, an alteration present in about 12% of ovarian tumors across various histological types (3). Mucinous cystadenomas, considered precursors of ovarian cancer, are primarily benign neoplasms characterized by mucin-producing epithelial cells (4). The progression from benign mucinous ovarian tumors to carcinomas is thought to occur due to the accumulation of multiple genetic abnormalities, resulting in the progression from benign to borderline to carcinoma (5). We were not able to determine if the *PIK3CA* variant identified by CancerSEEK originated from the ovarian mucinous cystadenoma. However, intratumor genetic heterogeneity is common in mucinous ovarian tumors. *BRAF* and *p53* mutations are frequently identified within mucinous borderline tumor components, while *KRAS* and *PIK3CA* mutations are more commonly identified within the mucinous ovarian carcinoma components of mucinous ovarian tumors (5, 6). Among post-menopausal women, the preferred treatment options are salpingo-oophorectomy, as was the approach in this patient, or ovarian cystectomy (4).

The diagnosis of appendiceal carcinoma *in situ* followed the detection of a variant in the *TP53* gene. The inactivation of tumor suppressor genes, such as *TP53* and *SMAD4*, along with the activation of oncogenes, such as *KRAS*, *GNAS*, and *BRAF*, are thought to drive appendiceal tumor progression though proliferation, angiogenesis, and evasion of apoptosis (7). However, molecular analysis of this participant’s paraffin embedded tissue sample did not confirm the presence of the *TP53* variant, possibly due to intratumoral heterogeneity. Low-grade appendiceal mucinous neoplasm (LAMN) is a rare appendiceal tumor identified in fewer than 0.3% of appendectomy specimens and generally presents as an incidental finding (8). While the risk of invasion with a LAMN is low, it may rupture and seed mucin and neoplastic cells into the peritoneum.

In the third case, the CancerSEEK test identified a variant in the *KRAS* gene. *KRAS* variants are common in both colorectal pre-cancer and cancer (9–11), with reported *KRAS* mutation frequencies of 32.4% in adenomas and 45.5% of colorectal carcinomas (12). The diagnostic evaluation revealed large (4.5 cm and 5.0 cm) colon adenomas with high-grade dysplasia, which have high likelihood of progressing to invasive colorectal cancer (13).

The DETECT-A study was the first prospective interventional trial to evaluate an MCED blood test, and the objectives included investigating the study feasibility and participant safety. Several safety measures were implemented that may have contributed to the turnaround time for study results. Initially, a baseline CancerSEEK test was performed, followed by a confirmatory test for those participants with positive baseline tests to rule out mutations attributed to CHIP. The median time from the 1^st^ blood draw to result was 4.8 months and from the 2^nd^ blood draw to return of result was 2.3 months. Subsequently, the multidisciplinary review committee assessment took place, and PET-CT imaging was carried out. Additional procedures, including a lung CT scan for the first participant and colonoscopies for the other two, also contributed to the delay before surgery. The details of these additional procedures may be found in Lennon, et al. *Science*, 2020) (1). Future tests implemented into clinical practice will require much more rapid turnaround times with optimized clinical workflows.

## 5 Conclusion

These three cases illustrate examples of pre-cancerous conditions that may be diagnosed consequent to a work-up following a positive cancer signal from an MCED test. While the known biology of such lesions suggests that they could have plausibly been the source of the somatic variants that triggered the MCED results, we were not able to confirm the relationship in these 3 cases and therefore cannot rule out the possibility that these were incidental findings on PET-CT imaging. Future studies should examine these questions further by evaluating the biology of excised lesions and performing MCED tests after resection to determine if the signal had resolved. Investigators planning MCED studies should consider these uncommon outcomes in their study plans, and patients and clinicians need to be prepared to encounter some cases with pre-cancerous lesions.

## Data Availability

The raw data supporting the conclusions of this article will be made available by the authors, without undue reservation.
